# Dietary Calcium to Digestible Phosphorus Ratio for Optimal Growth Performance and Bone Mineralization in Growing and Finishing Pigs

**DOI:** 10.3390/ani10020178

**Published:** 2020-01-21

**Authors:** Patrick Schlegel, Andreas Gutzwiller

**Affiliations:** Agroscope, Swine Research Unit, 1725 Posieux, Switzerland

**Keywords:** pig, calcium, phosphorus, zinc, phytase, dual-energy X-ray absorptiometry, DXA

## Abstract

**Simple Summary:**

Minimizing the use of dietary non-renewable mineral phosphates improves the sustainability of phosphorus use in growing-finishing pigs. As the phosphorus metabolism is closely linked to calcium, this experiment compared three dietary calcium to digestible phosphorus ratios within two phosphorus levels to determine the optimum levels for growth performance and bone mineralization. The lowest calcium level was insufficient for an efficient metabolic use of P, the medium level was sufficient to maximize growth performance and highest level further improved bone mineralization. The low digestible phosphorus level enabled a complete removal of supplemented mineral phosphates in the finisher period, which resulted, per pig, in a decrease of its use by 65% and a decrease of the calculated P excretion by 41%, without impaired growth performance. However, a reduced bone mineralization was observed. This study shows that there is potential to reduce the digestible phosphorus compared to practiced levels in grower–finisher pigs and shows that the optimal calcium to digestible phosphorus ratio needs to be maintained at the upper range of the actually available recommendations.

**Abstract:**

Within the context of maximizing the use of dietary phosphorus, a growing-finishing pig study was conducted to determine the optimal total dietary calcium (Ca) to digestible phosphorus (dP) ratio and to verify the possibility of mineral phosphate removal during the finishing period on growth performance and mineral status. The potential for replacing chemical and mechanical bone properties by dual energy X-ray absorptiometry (DXA) measures on non-dissected feet was also verified. Three Ca to dP ratios (2.2:1, 2.5:1 and 2.8:1) within two dP levels (P+, P–) were fed during 91 days to 84 pigs. The grower and finisher P+ diets contained 3.0 and 2.4 and P– diets contained 2.5 and 1.7 g dP/kg, respectively. Growth performance and blood serum mineral content were independent of treatments, except that 2.2:1 impaired finisher feed conversion ratio compared to 2.5:1 and 2.8:1. Urinary P concentration increased by 37% in 2.2:1 compared to 2.5:1 and 2.8:1. Maximal load on bone and DXA mineral density were reduced in 2.2:1 compared to 2.8:1. Bone ash and volumetric density were reduced in 2.2:1 and 2.5:1 compared to 2.8:1. Diet P– reduced bone ash, maximal load, volumetric density and DXA bone mineral content and density. No interaction was observed between Ca and dP level. Therefore, 2.2:1 was insufficient for an efficient metabolic use of P, 2.5:1 was sufficient to maximize growth performance and 2.8:1 further improved bone mineralization. Increasing dietary Ca did not impair bone zinc content. Diets P– without supplemented mineral phosphates during the finisher period resulted, per pig, in a decrease of its use by 65% and of the calculated P excretion by 41%, without impaired growth performance. Finally, DXA data responded to dietary treatments as did labor intensive chemical and mechanical bone properties.

## 1. Introduction

A sustainable use of dietary phosphorus (P) is necessary to minimize its excretion and to limit the use of non-renewable mineral phosphates being progressively a limiting source of P [1]. This, while animal growth performance needs to be fully expressed. The digestive and metabolic processes of P are complex as they are closely linked with those of calcium (Ca) [2]. Excessive dietary Ca may limit P absorbability as well as zinc (Zn) bioavailability, whereas insufficient dietary Ca limits bone formation and, in consequence, retained P [3,4]. Therefore, when aiming for an efficient use of P, dietary Ca is considered and usually expressed as a total Ca to digestible P ratio or a digestible Ca to digestible P ratio. This ratio was recently investigated for maximal growth performance and maximal bone mineralization within a relatively wide range of Ca and P levels in growing-finishing pigs [5,6,7]. The actual recommended total Ca and apparent total tract digestible P (dP) result to Ca to dP ratios fluctuating between 2.3:1 and 3.0:1 for grower–finisher pigs [8,9,10,11]. Within this Ca to dP range, the Ca supply can thus vary by ± 15% for a given dP level. It remains open if, within this limited range of Ca to dP ratio, the efficiency of the P metabolic use is impacted. Finally, additional data from Ca dose-response studies are helpful to fine-tune dietary Ca and P models for growing pigs being currently developed.

Parameters used to assess the physiological response to modified dietary Ca and P need to be highly sensitive, easily collected and cost-effective. Parameters of bone integrity for mineralization and urinary mineral content are considered as highly sensitive and directly affected by the homeostatic control of Ca and P. Bone ash content and mechanical tests on bones, such as volumetric density or breaking strength are commonly used for assessing bone integrity for mineralization and risk of fracture. Dual energy X-ray absorptiometry (DXA) technology has progressively been adopted to assess body composition in growing pigs [12,13]. It represents an alternative non-invasive technology to easily and rapidly assess bone mineral content (BMC) and density (BMD) from selected bones [14,15,16]. Thus, the DXA BMC and BMD from non-dissected easily accessible animal part, such as the front foot may be an alternative to chemical and mechanical tests from dissected bones.

In this context, the aims of this experiment were 1) to measure growth performance and Ca, P and Zn status of pigs between 20 and 100 kg BW fed a Ca to dP ratio of 2.2:1, 2.5:1, and 2.8:1 in either a P-adequate or marginal diet; 2) to evaluate the response of BMC and BMD by DXA from a non-dissected foot and their accuracy to chemical and mechanical properties of dissected bones.

## 2. Materials and Methods

### 2.1. Experimental Diets

Six grower (optimized for 40 kg BW) and finisher (optimized for 80 kg BW) diets (Table 1) were formulated according to current Swiss feeding recommendations for pigs [8], except for Ca and digestible dP. Grower diets were formulated to contain 2.5 (marginal, P–) or 3.0 (recommended, P+) g dP/kg and to contain Ca with Ca:dP ratios of 2.2:1, 2.5:1, or 2.8:1. Finisher diets were formulated to contain 1.7 (marginal, P−) or 2.4 (recommended, P+) g dP/kg and to contain Ca with Ca:dP ratios as in the grower diets. Microbial phytase (Natuphos^®^ 5000G, BASF, Ludwigshafen, Germany, equivalency of 0.80 g digestible P per 500 FTU) was included in all diets at 500 FTU/kg diet. The P content was modified with the inclusion of monocalcium phosphate (Protector S.A., Lucens, Switzerland, analyzed 175.8 g Ca, 189.4 g P) and the Ca content was modified with the inclusion of CaCO_3_ (Holcim S.A., La Sarraz, Switzerland, analyzed 391.1 g Ca/kg). To maintain digestible energy and crude protein content in all grower and finisher diets, inclusion levels of wheat starch and fat were adapted. Each experimental diet included differently colored maize cob pellets to allow visual identification. Diets were produced at the Agroscope experimental feed mill and were pelleted (60 °C, 4 mm diameter). The software Allix2 (A-Systems S.A., Versailles, France) was used to formulate the experimental diets, based on the analyzed dry matter (DM), crude protein, crude fiber, crude fat, ash, Ca and P concentrations of each feedstuff. The other mineral concentrations, digestible amino acid profiles and coefficients for digestible P of each feedstuff were formulated according to the open-access Swiss national reference table values [17].

### 2.2. Animals and Experimental Procedures

The experimental procedure was approved by the Office for Food Safety and Veterinary Affairs (approvals n° 2012_57_FR and 2015_02_FR). In two series, a total of 84 Large White pigs (initial BW of 22.8 ± 2.2 kg (mean ± SD) and 23.7 ± 2.2 kg in series 1 and 2, respectively) were selected from the Agroscope herd and blocked by litter, BW and gender (female and castrated males). Each pig from a block (12 blocks with 3 pigs per block in Series 1, 12 blocks with 4 pigs per block in Series 2) was randomly allocated to a treatment. Series 1 consisted of three treatments (P+2.2:1, P+2.5:1, P+2.8:1 with 12 pigs per treatment) and series 2 consisted of four treatments (P+2.5:1, P–2.2:1, P−2.5:1, P−2.8:1 with 12 pigs per treatment). All animals within a series were housed in one pen (95 m^2^) equipped with four computer controlled feeding stations (Schauer Agrotronic GmbH, Prambachkirchen, Austria) as described previously by Bee et al. [18]. The grower and the finisher diets were offered ad libitum from d 0 to 42 and from d 42 to 91, respectively. Pigs had free access to one of the feeding station and to water. Access to feed was stopped the day before slaughter at 17h00. The next morning (d 91), pigs were anaesthetized using CO_2_ followed by exsanguination at the Agroscope research abattoir located on the experimental site. 

### 2.3. Data and Sample Collection

Feed intake (FI) was recorded at each individual visit and totalized per pig on a daily basis. Pigs were weighed on days 0, 42, and 91. Water was sampled in a previous experiment and its concentration was 66 and <2 mg/L of Ca and P, respectively. Samples of the grower and finisher diets were collected weekly, pooled per treatment and feed type and milled using a 1.0 mm screen (Brabender, Duisburg, Germany) before analysis. A blood sample from each pig was collected on d 42 in the morning from the jugular vein and d 91 at exsanguination. Blood samples (9 mL) were centrifuged (3000× *g*, 15 min) within 2 h after collection and serum was stored at −20 °C until analysis. A urine sample was collected at evisceration from the urinary bladder of castrated males and frozen at –20 °C for further analysis. The front feet were cut at the middle carpal joint. The metacarpus (Mc) III and IV from the right foot were dissected within half a day after slaughter. The left foot and the right Mc’s were stored in sealed plastic bags at −20 °C. The Mc IV was transferred from the freezer to a refrigerator 12 h before use. Their longest mid-shaft diameter was measured and the mid-section area was calculated as π * (longest mid-shaft diameter/2)^2^. Their maximal load until bone breaking was then determined using the three-point bending test (ZwickiLine Material-Prüfmaschinen Z2.5TN, Zwick Roell, Ulm, Germany). The bones were held by two supports spaced 43 mm apart and were broken by a wedge lowered on the center of the bone at a speed of 2 mm/s and a maximal pressure of 2500 N. The force was measured by a pressure-sensitive cell, and peaks of maximum force were recorded. The Mc III and IV were then autoclaved (121 °C, 1 bar, 45 min) and cleaned of soft tissues. The Mc III were weighed in air and suspended in distilled water to calculate the volumetric bone density. The autoclaved Mc were then crushed, defatted with acetone, dried overnight at 60 °C, weighed and milled using a 1.0 mm screen (Grindomix Retsch, Haan, Germany) before chemical analysis. The left foot was scanned on dorso-palmar plane orientation for bone mineral content (BMC), bone surface area and bone mineral density (BMD) by dual-energy X-ray absorptiometry on GE Healthcare i-DXA (GE Medical Systems, Glattbrugg, Switzerland) using the Small Animal software program on enCORE^TM^ version 16 (100 kV, 0.188 mA). The DXA was available only after completion of the experiment, reason for having used the left foot stored for this occasion. Three regions of interest were defined during image processing of the scans: the foot from the carpo-metacarpal joint comprising metacarpal bones and phalanges, the Mc III and the Mc IV.

### 2.4. Chemical Analysis of Samples

Dry matter content was quantified thermo-gravimetrically by heating at 105 °C for 3 h and ash content was subsequently determined after incineration at 550 °C until constant weight was attained (prepAsh, Precisa Gravimetrics AG, Dietikon, Switzerland). Crude protein content in diets was determined as 6.25 × nitrogen, where nitrogen content was determined using an automated analyzer (TruMac CNS, Leco, Mönchengladbach, Germany). Dry ashed feed and Mc were solubilized with nitric acid and their mineral concentration was analyzed according to the European Standard EN 155510:2008 using an inductively coupled plasma optical emission spectrometer (ICP-OES, Optima 7300 DV, Perkin-Elmer, Schwerzenbach, Switzerland). Phytic acid content and phytase activity in diets were determined as previously described by Schlegel and Gutzwiller [19]. The serum and urine samples were assayed using commercially available kits according to manufacturer’s instruction on a BT1500 autoanalyzer (Biotecnica Instruments S.p.A., Roma, Italy) to determine, creatinine (Biotecnica Instruments S.p.A., Roma, Italy), inorganic Ca and inorganic phosphate (Greiner Diagnostic GmbH, Langenthal, Switzerland) concentrations. All analyses were performed in the accredited Agroscope laboratories in duplicate, except DM and ash as single analysis.

### 2.5. Statistical Analysis

The individual pig served as the experimental unit, thus providing 24 replicates for P+2.5:1 and 12 replicates for the other treatments. Data were analyzed using the mixed model procedure of SYSTAT 13 software (Systat Software GmbH, Erkrath, Germany). The statistical model included series (1,2), dP level (P+, P−), Ca:dP ratio (2.2:1, 2.5:1, 2.8:1), and dP level × Ca:dP ratio interaction as fixed effects and block as random effect. Comparisons among means were calculated using the post-hoc Tuckey test. The BMC and BMD of Mc III and Mc IV areas from the non-dissected left foot were compared with Mc ash weight, Mc III volumetric density and maximal load on Mc IV using Pearson correlation coefficients and a linear model regression: Y_i_ = α + β X + e_i_, where α is the intercept, β the slope. Differences were considered significant when *p* < 0.05 and trends were noted between *p* = 0.05 and 0.10.

## 3. Results

### 3.1. Diets

The analyzed and calculated nutrient values of the experimental diets are presented in Table 2. The analyzed Ca concentrations increased from diets 2.2:1 to 2.8:1 and were lower in P– diets than in P+ diets. Calcium from mineral sources represented between 60% and 75% of total dietary Ca and P from mineral sources represented between 0 and 36% of total dietary P. The analyzed phytic P content was the same within the grower and finisher diets, respectively. The analyzed mean phytase activity was 765 and 919 FTU/kg in the grower and finisher diets, respectively, which represent the sum of the vegetal phytase activity and the added microbial phytase activity.

### 3.2. Growth Performance

Three pigs (1 from P+2.5:1 and 2 from P−2.5:1) died on d 42, 56 and 84, respectively. The reasons are apparently not related to the dietary treatments as two of them were diagnosed with intestinal torsion. No series effect and no dP × Ca:dP ratio interaction were observed (*p* > 0.05) on animal performance values, thus main effects are presented. The obtained growth performance (Table 3) was comparable to the one generally obtained from the herd. Growth performance was not affected by the dP level, except for a trend for greater (*p* < 0.10) daily BW gain in the finisher period when P− was fed compared to P+. The Ca:dP ratio of 2.2:1 impaired (*p* < 0.01) feed conversion ratio (FCR) by 3.5% in the finisher period and tended (*p* < 0.10) to be impaired by 1.7% in the overall period compared to the other Ca:dP ratios.

### 3.3. Urinary and Blood Serum Concentrations and Bone Traits

No series effect and no digestible P × Ca:dP interaction were observed (*p* > 0.05) in urinary and serum values and in bone traits. Urinary pH and Ca concentrations were not affected (*p* > 0.05) by dietary treatments and urinary P concentration was increased (*p* < 0.01) by 37% when 2.2:1 was fed compared to the other Ca:dP ratios (Table 4). Blood serum Ca and P on d 42 and on day of slaughter were not affected (*p* > 0.05) by dietary treatments. The Mc defatted bone weights, the mid-section surface, the volume and the DXA bone areas were not affected (*p* > 0.10) by dietary treatments. Diets P+ increased (*p* < 0.05) Mc ash, Ca and P content, volumetric density, maximal load, BMC and BMD (Table 5). Mc ash content and volumetric density were increased (*p* < 0.05) when 2.8:1 was fed compared to 2.2:1 and 2.5:1. In addition, maximum load and BMD on Mc were increased (*p* < 0.05) and BMC of the foot tended (*p* < 0.10) to be increased with diet 2.8:1 compared to 2.2:1. Mc Zn concentrations were independent (*p* > 0.05) from dietary treatments. The Pearson correlations and linear relationships between BMD and BMC of non-dissected Mc of the foot and Mc ash weight, volumetric density and maximal load are presented in Table 6. Pearson correlations were between 0.51 and 0.91 and the slopes of the linear regressions were all different from 0 (*p* < 0.01) with R^2^ values between 0.26 and 0.81.

## 4. Discussion

### 4.1. Effect of The Digestible Phosphorus Levels

The formulated dP levels were comparable to the estimated dP concentrations using the analyzed values of P, phytic P, phytase activity and Ca according to the regression provided by Létourneau-Montminy et al. [3]. The total P concentration in the grower and finisher P+ diets agree with the current Swiss feeding dP recommendations for growing-finishing pigs [8] and are representative for commercial Swiss diets supplemented with phytase [20]. The grower and finisher P– diets contained 16% and 29% less dP than the respective P+ diets enabling a complete removal of supplemental mineral phosphates in the finisher diets and permitting a 65% lower total mineral phosphate consumption. Mineral phosphates are non-renewable resources and the market prices of quality phosphates, low in heavy metal concentrations, are expected to increase with increasing mining costs of the raw material [1]. The total P excretion (P intake – P gain in BW) from 23 to 103 kg BW, was estimated at 516 and at 303 g P per pig fed the P+ and P– diets, respectively when using a mean P retention of 5.32 g per kg BW gain, obtained from the allometric empty BW regression by Ruiz-Ascacibar et al. [21]. The P– diets, thus permitted to reduce P excretion per pig by 213 g P (41%) compared to the P+ diets without detrimental effect on growth performance, blood serum mineral and urinary P content. Such a reduction in P excretion per pig is relevant when farm based nutrient cycles are mandatory, such as in Switzerland since 1998 [22]. Comparable reductions of dP levels in other studies [23,24,25] comply with the present results for not having limited the growth performance of pigs. But, further reduced mean dP levels in a grower–finisher diet (1.4 g dP/kg) limited growth performance [26]. However, the P– diets from the present study appeared not to be sufficient to maximize bone mineralization when reaching slaughter weight.

### 4.2. Effect of The Calcium Levels

The present data indicate that a Ca:dP ratio of 2.2:1 was insufficient for an optimal physiological use of P illustrated by an increased urinary P and a reduced bone ash concentration, volumetric bone density, maximal load and BMD as compared to Ca:dP ratios of respectively 2.5 and 2.8:1. The increase in urinary P concentration indicates that absorbed P could not be used for hydroxyapatite formation as available Ca was lacking or that hydroxyapatite was mobilized from the bone to release Ca and consequently P. Both cases are indicative for Ca deficiency, which would finally lead to a limited bone mineralization [3,5,27,28]. At the end of the grower and finisher periods, blood serum Ca and P were not affected by dietary Ca levels, suggesting that the low dietary Ca was not yet limiting for circulating minerals in the metabolism. The reason for the impaired FCR by 4% solely observed during the finisher period with a Ca:dP ratio of 2.2:1 compared to the other ratios was related to the numerically 4% impaired daily BW gain, especially observed in the P+2.2:1 diet (909 g/d). According to the regressions by Lagos et al. [6] the BW gain between 50 and 80 kg BW was impaired as well with lower Ca levels in diets, which met P requirement.

The present data indicate that the diets with Ca:dP ratios of respectively 2.5 and 2.8:1 were adequate for growth and sufficient for an optimal use of P, whereas 2.8:1 permitted to improve bone mineralization (especially ash concentration, volumetric density). An excess in dietary Ca can lead to increased urinary Ca and depressed serum P levels [5,19], thus resulting in impaired dietary P efficiency. This negative effect was not observed with 2.8:1, suggesting that higher Ca:dP ratios are required to limit the dietary use of P.

Excessive dietary Ca may also impair the bioavailability of Zn as initially described by Oberleas et al. [4], who fed 8 g Ca/kg diet in a semi-synthetic diet including sodium phytate to rats. Supplemented Ca stabilizes the phytic acid to Zn complexation in the digestive tract when sodium phytate and Zn are added to the diet. In cereal-based diets fed to growing pigs, a moderate Ca excess had however limited or inexistent antagonistic effects [29,30,31] on apparent total tract digestibility of Zn unless extremely high Ca levels (15 g/kg) were applied which resulted to reduced liver Zn and serum alkaline phosphatase activity [32]. The present data indicate that dietary Ca did not affect bone Zn concentration, which is a sensible parameter in case of a decreasing Zn status [33]. The limited or non-observed antagonism of supplemental Ca on Zn bioavailability supports the conclusions made that vegetal phytates are bound to cations, such as Ca and Zn from the plant, and thus do not interact with supplemental Zn to modify Zn bioavailability [33,34]. This would also be the case for supplemented Ca. Thus, as previously observed in post-weaning piglets [19], dietary Zn recommendations do not need to be adapted according to dietary Ca levels susceptible to be realistic in pig diets. 

### 4.3. Potential of Dual Energy X-Ray Absorptiometry Technology without Bone Dissection to Replace other Bone Mineralisation Traits

Dual energy X-ray absorptiometry technology enables a labor friendly and rapid measurement of bone mineralization characteristics expressed as BMC and BMD. Previous studies have shown that the linear relationship between BMC or BMD and chemical (ash) and mechanical properties (volumetric density, maximal load) of dissected bones were acceptable to excellent [14,15,16]. In the present study, DXA measures from a non-dissected foot were compared with the chemical and mechanical properties of dissected Mc to verify if the labor for dissection, chemical analysis, and physical measures can be saved. This was clearly the case, as not only the dietary treatment effect on BMC and BMD from the selected foot areas were highly sensitive and comparable with Mc ash weight, ash content, volumetric density and maximal load. The coefficients of variability (SEM/mean of the least square means * 100) of BMC and BMD ranged between 1.63% and 2.93% which were comparable with those of Mc ash weight, volumetric density and maximal load (3.09%, 1.03%, and 4.06%, respectively). These findings are in line with the effect of varying dietary P on BMD from a foot compared to volumetric density and ash concentration of dissected metatarsus [25]. The present results thus show that DXA measurements on selected areas from a non-dissected foot can be used to replace chemical and mechanical traits of Mc’s when studying Ca and P nutrition in growing pigs. The accuracy to estimate Mc ash weight using BMC from the Mc area of a non-dissected foot was fairly good with an R^2^ of 0.81. However, the accuracy to estimate maximal load and volumetric density was more critical. Measures with DXA on a non-dissected front foot induce a partial overlap of Mc III with Mc II and of Mc IV with Mc V within the two dimensional region of interest. As this is not the case for the chemical and mechanical properties of dissected Mc’s, this may have contributed to limit the R^2^ values. In addition, the fact that BMD is expressed as BMC divided by the projected two-dimensional area of the bone, whereas the volumetric density accounts for bone volume may also have contributed to limit these R^2^ values. Although, the Pearson relations between in vivo BMC and ash weight and in vivo BMD and volumetric density from non-overlapping bones such as vertebrae [14] were not any better than the present ones and no difference in BMD, BMC and breaking strength from Mc was observed between the left and right front foot from pigs [16].

## 5. Conclusions

Within a limited range of dietary Ca to digestible P ratio, the metabolic use of P was affected, but not the bioavailability of Zn. To maximize the use of dietary P by the growing-finishing pig, this ratio should not be below 2.5:1. This study also showed that growth performance was maintained with a reduced level of digestible P allowing a complete removal of mineral phosphates between 60 and 100 kg body weight. Finally, the assessment of DXA bone mineral content and density of non-dissected feet represents an alternative to the labor intensive chemical and mechanical bone properties.

## Figures and Tables

**Table 1:** Ingredients in experimental diets (g/kg).

Period	Grower						Finisher					
Digestible P Level ^1^	P−			P+			P−			P+		
Ca:Digestible P Ratio	2.2:1	2.5:1	2.8:1	2.2:1	2.5:1	2.8:1	2.2:1	2.5:1	2.8:1	2.2:1	2.5:1	2.8:1
Barley	575	575	575	575	575	575	504	504	504	504	504	504
Wheat	179	179	179	179	179	179	246	246	246	246	246	246
Oats							10	10	10	10	10	10
Wheat bran							1.6	1.6	1.6	1.6	1.6	1.6
Expelled soybean meal	6.3	6.3	6.3	6.3	6.3	6.3	89	89	89	89	89	89
Potato protein	94	94	94	94	94	94						
Sugarbeet pulp dehydr.	83	83	83	83	83	83	86	86	86	86	86	86
Wheat starch	29.2	26.0	23.2	21.8	17.8	14.0	39.2	37.3	35.0	32.0	29.0	25.8
Fat (tallow and lard)	0.6	2.0	3.2	3.8	5.6	7.2		0.6	1.6	2.8	4.1	5.6
L-Lysine-HCl	3.2	3.2	3.2	3.2	3.2	3.2	2.3	2.3	2.3	2.3	2.3	2.3
DL-Methionine	0.7	0.7	0.7	0.7	0.7	0.7						
L-Threonine	1.1	1.1	1.1	1.1	1.1	1.1	0.9	0.9	0.9	0.9	0.9	0.9
L-Tryptophane	0.4	0.4	0.4	0.4	0.4	0.4						
Calcium carbonate	7.6	9.4	11.0	9.0	11.2	13.4	5.5	6.8	8.1	6.8	8.5	10.2
Monocal. phosphate	3.6	3.6	3.6	6.4	6.4	6.4				3.1	3.1	3.1
Sodium chloride	3.2	3.2	3.2	3.2	3.2	3.2	2.4	2.4	2.4	2.4	2.4	2.4
Vit. and min. premix ^2^	4.0	4.0	4.0	4.0	4.0	4.0	4.0	4.0	4.0	4.0	4.0	4.0
Binder ^3^	3.0	3.0	3.0	3.0	3.0	3.0	3.0	3.0	3.0	3.0	3.0	3.0
Col. maize cob pellets ^4^	6.0	6.0	6.0	6.0	6.0	6.0	6.0	6.0	6.0	6.0	6.0	6.0
Microbial phytase ^5^	0.1	0.1	0.1	0.1	0.1	0.1	0.1	0.1	0.1	0.1	0.1	0.1

^1^ P− = Formulated to contain 2.5 and 1.7 g digestible P/kg grower and finisher diets, respectively. P+ = Formulated to contain 3.0 and 2.4 g digestible P/kg grower and finisher diets, respectively. ^2^ Supplied per kg of diet: 4 mg Cu; 20 mg Fe; 10 mg Mn; 45 mg Zn; 0.15 mg I; 0.15 mg Se; 4000 IU vitamin A; 400 IU vitamin D_3_; 200 mg choline; 2 mg vitamin B_1_; 3 mg vitamin B_2_; 3 mg vitamin B_6_; 15 mg niacin; 0.02 mg vitamin B12; 15 mg pantothenic acid; 0.05 mg biotin; 0.5 mg folic acid; 65 mg vitamin E; 1 mg vitamin K_3_. ^3^ Pellan, Mikro-Technik, Bürgstadt, Germany. ^4^ Mikrogrit, Microtracers, San Francisco, U.S.A. ^5^ Natuphos^®^ 5000G, BASF, Ludwigshafen, Germany, 5000 FTU/g, equivalency of 0.80 g digestible P/500 FTU.

**Table 2:** Analyzed and calculated nutrient values in experimental diets.

Period	Grower					Finisher						
Digestible P Level ^1^	P−			P+		P−						P+
Ca:Digestible P Ratio	2.2:1	2.5:1	2.8:1	2.2:1	2.5:1	2.8:1	2.2:1	2.5:1	2.8:1	2.2:1	2.5:1	2.8:1
Dry matter (g/kg) ^2^	896	899	898	887	891	888	894	892	893	884	889	886
Dig. energy (MJ/kg) ^3^	13.0	13.1	13.2	13.1	13.1	13.1	13.0	12.9	13.3	12.9	12.9	12.8
Crude protein (g/kg) ^2^	164	165	165	156	160	158	127	127	128	130	129	128
Ash (g/kg) ^2^	37	39	40	42	44	47	34	35	37	40	40	43
Dig. lysine (g/kg) ^3^	9.2	9.2	9.2	9.2	9.2	9.2	6.2	6.2	6.2	6.2	6.2	6.2
Dig. methionine (g/kg) ^3^	3.2	3.2	3.2	3.2	3.2	3.2	1.6	1.6	1.6	1.6	1.6	1.6
Dig. threonine (g/kg) ^3^	6.1	6.1	6.1	6.1	6.1	6.1	4.1	4.1	4.1	4.1	4.1	4.1
Ca (g/kg) ^2^	5.3	5.9	6.5	6.6	7.2	8.3	3.8	4.4	4.8	5.1	5.7	6.3
P (g/kg) ^2^	3.9	3.7	3.9	4.4	4.3	4.4	2.9	3.0	2.9	4.1	3.9	4.3
Phytic P (g/kg) ^2^	2.1	2.0	2.0	1.8	2.0	2.1	2.6	2.4	2.5	2.3	2.4	2.2
Non phytic P (g/kg) ^3^	1.8	1.7	1.9	2.6	2.3	2.3	0.4	0.6	0.4	1.8	1.4	2.1
Phytase activity (U/kg) ^2^	746	722	704	794	775	850	919	895	877	993	991	837
Digestible P (g/kg) ^3^	2.5	2.5	2.5	3.0	3.0	3.0	1.7	1.7	1.7	2.4	2.4	2.4
Ca:dP ^3^	2.1	2.4	2.6	2.2	2.5	2.8	2.2	2.5	2.8	2.1	2.4	2.6
Zn (mg/kg) ^2^	67	65	69	69	65	69	67	70	67	75	70	77

^1^ P– = Formulated to contain 2.5 and 1.7 g digestible P/kg grower and finisher diets, respectively. P+ = Formulated to contain 3.0 and 2.4 g digestible P/kg grower and finisher diets, respectively. ^2^ Analyzed as described under chapter Material and Methods. ^3^ Calculated.

**Table 3:** Effect of digestible P (g/kg) and Ca:digestible P ratio on growth performance in grower (d 0 to 42) and in finisher (d 42 to 91) pigs.

Item	Digestible P ^1^			Ca:Digestible P				
	P−	P+	*p*-Value ^2^	2.2:1	2.5:1	2.8:1	*p*-Value ^2^	SEM
N°	36	48		24	36	24		
BW, Initial (kg)	23.2	23.2	n.s.	23.1	23.2	23.3	n.s.	0.58
BW, d 42 (kg)	57.0	58.9	n.s.	58.5	57.7	57.6	n.s.	1.29
BW, final (kg)	103.3	102.9	n.s.	102.8	103.4	103.0	n.s.	2.05
Carcass weight (kg)	82.7	82.3	n.s.	82.1	82.8	82.5	n.s.	1.77
BW gain grower (g/d)	827	866	n.s.	857	844	838	n.s.	22.3
BW gain finisher (g/d)	978	924	+	927	965	962	n.s.	24.6
BW gain overall (g/d)	898	893	n.s.	893	899	894	n.s.	20.8
FI grower (g/d)	1807	1880	n.s.	1867	1840	1824	n.s.	61.4
FI finisher (g/d)	3101	2951	n.s.	3015	3058	3005	n.s.	96.4
FI overall (g/d)	2395	2362	n.s.	2395	2387	2353	n.s.	73.8
FCR grower (g/d)	2.18	2.17	n.s.	2.17	2.18	2.17	n.s.	0.036
FCR finisher (g/d)	3.17	3.19	n.s.	3.25 ^b^	3.16 ^a^	3.12 ^a^	**	0.052
FCR overall (g/d)	2.66	2.64	n.s.	2.68	2.65	2.62	+	0.038

^1^ P– = Formulated to contain 2.5 and 1.7 g digestible P/kg grower and finisher diets, respectively. P+ = Formulated to contain 3.0 and 2.4 g digestible P/kg grower and finisher diets, respectively. ^2^ ** *p* < 0.01; n.s. (non-significant) *p* > 0.05; + *p* < 0.10; two-way interactions were not significant. Thus, only main effects are presented. For each main factor, values in the same row not followed by the same letter differ.

**Table 4:** Effect of digestible P (g/kg) and Ca:digestible P on blood serum and urinary concentrations in grower pigs on d 42 and in finisher pigs on d 91.

Item	Digestible P^1^			Ca:Digestible P				
	P–	P+	*p*-Value ^2^	2.2:1	2.5:1	2.8:1	*p*-Value ^2^	SEM
N°	36	48		24	36	24		
Urine at slaughter ^3^								
pH	7.53	7.56	n.s.	7.55	7.51	7.58	n.s.	0.084
Creatinine (mmol/L)	7.27	8.05	n.s.	7.78	7.22	7.98	n.s.	1.141
Ca (mol/mol creatinine)	0.07	0.09	n.s.	0.08	0.09	0.08	n.s.	0.021
P (mol/mol creatinine)	1.09	1.24	n.s.	1.42^a^	1.00^b^	1.07^b^	**	0.146
Blood serum								
Ca (mmol/L) on day 42	2.88	2.85	n.s.	2.85	2.86	2.89	n.s.	0.030
P (mmol/L) on day 42	2.53	2.49	n.s.	2.53	2.52	2.46	n.s.	0.043
Ca (mmol/L) at slaughter	2.92	2.88	n.s.	2.87	2.94	2.89	n.s.	0.043
P (mmol/L) at slaughter	3.10	3.08	n.s.	3.13	3.03	3.12	n.s.	0.075

^1^ P– = Formulated to contain 2.5 and 1.7 g digestible P/kg grower and finisher diets, respectively. P+ = Formulated to contain 3.0 and 2.4 g digestible P/kg grower and finisher diets, respectively. ^2^ ** *p* < 0.01; * *p* < 0.05; n.s. (non-significant) *p* > 0.05; two-way interactions were not significant. Thus only main effects are presented. For each main factor, values in the same row not followed by the same letter differ. ^3^ Data from 49 samples in total (7 per treatment, except 14 in P+2.5:1)

**Table 5:** Effect of digestible P (g/kg) and Ca:digestible P ratio on bone traits in pigs on d 91.

Item	Digestible P^1^			Ca:Digestible P				
	P–	P+	*p*-Value ^2^	2.2:1	2.5:1	2.8:1	*p*-Value ^2^	SEM
N°	36	48		24	36	24		
Metacarpus III and IV								
Weight, defatted (g DM)	30.6	32.0	n.s.	30.8	31.6	31.6	n.s.	0.95
Ash (g)	16.9	18.3	*	17.3	17.7	17.9	n.s.	0.54
Ash (g/kg DM)	555^y^	573^x^	***	561^b^	559^b^	570^a^	*	4.1
Ca (g/kg DM)	207^y^	218^x^	**	215	210	213	n.s.	3.3
P (g/kg DM)	98^y^	102^x^	*	101	99	100	n.s.	1.8
Zn (mg/kg DM)	114	118	n.s.	116	116	117	n.s.	4.2
BMC (g) ^3^	17.1	18.6	*	17.3	18.0	18.2	n.s.	0.49
Bone area (cm^2^)	22.8	22.9	n.s.	22.7	23.2	22.8	n.s.	0.41
BMD (g/cm^2^) ^3^	0.754	0.806	***	0.763^b^	0.779^ab^	0.797^a^	**	0.014
Metacarpus III								
Weight (g)	22.0	23.1	n.s.	22.2	22.7	22.8	n.s.	0.56
Volume (cm^3^)	18.0	18.3	n.s.	18.0	18.4	18.1	n.s.	0.46
Density (g/cm^3^)	1.22^y^	1.26^x^	*	1.23^b^	1.23^b^	1.26^a^	*	0.013
BMC (g) ^3^	8.9	9.7	*	9.1	9.4	9.5	n.s.	0.26
Bone area (cm^2^)	11.6	11.7	n.s.	11.6	11.7	11.6	n.s.	0.21
BMD (g/cm^2^) ^3^	0.773	0.827	**	0.783^b^	0.801^ab^	0.816^a^	*	0.014
Metacarpus IV								
Mid-section area (mm^2^) ^4^	169	162	n.s.	168	165	164	n.s.	6.5
Maximal load (N)	1580^y^	1766^x^	**	1624^b^	1635^ab^	1757^a^	*	67.8
BMC (g) ^3^	8.2	8.8	*	8.3	8.6	8.7	n.s.	0.25
Bone area (cm^2^)	11.3	11.2	n.s.	11.1	11.4	11.2	n.s.	0.21
BMD (g/cm^2^) ^3^	0.734	0.785	**	0.743^b^	0.757^ab^	0.778^a^	*	0.015
Front Foot								
BMC (g) ^3^	38.8^y^	41.9^x^	*	39.2	40.7	41.1	+	0.99
Bone area (cm^2^)	62.9	65.0	n.s.	63.6	64.4	63.8	n.s.	1.11
BMD (g/cm^2^) ^3^	0.618^y^	0.644^x^	*	0.616^b^	0.632^ab^	0.644^a^	*	0.010

^1^ P– = Formulated to contain 2.5 and 1.7 g digestible P/kg grower and finisher diets, respectively. P+ = Formulated to contain 3.0 and 2.4 g digestible P/kg grower and finisher diets, respectively. ^2^ *** *p* < 0.001; ** *p* < 0.01; * *p* < 0.05; n.s. (non-significant) P > 0.10; + P < 0.10; two-way interactions were not significant. Thus, only main effects are presented. For each main factor, values in the same row not followed by the same letter differ. ^3^ BMC = bone mineral content; BMD = bone mineral density measured by DXA ^4^ Mid-section area = π * (longest mid-shaft diameter/2)^2.^

**Table 6:** Linear relationship between bone mineral content (BMC) and density (BMD) by dual energy X-ray absorptiometry from non-dissected foot and metacarpal (Mc) ash weight, volumetric density and maximal load.

Parameters		Intercept		Slope		Pearson		
DXA	Physical and Chemical	Coeff.	*p*-Value ^1^	Coeff.	*p*-Value ^1^	R^2^	SE	R
Mc III and IV BMD (g/cm^2^)	Mc III and IV ash weight (g)	-1.01	n.s.	24.08	***	0.52	1.404	0.71
Mc III and IV BMC (g)	Mc III and IV ash weight (g)	0.28	n.s.	0.96	***	0.81	0.867	0.91
Mc III BMD (g/cm^2^)	Mc III vol. density (g/cm^3^)	0.92	***	0.41	***	0.26	0.043	0.51
Mc IV BMD (g/cm^2^)	Mc IV max. load (N)	-201	n.s.	2502	***	0.57	151.8	0.75
Mc IV BMC (g)	Mc IV max. load (N)	283	n.s.	167	***	0.54	157.3	0.73

^1^*** *p* < 0.001; n.s. (non-significant).
